# Supplementary material for: Development and Validation of a Clinically Relevant Workflow for MR-Guided Volumetric Arc Therapy in a Rabbit Model of Head and Neck Cancer
Source: Cancers (Basel). 2020 Mar 1;12(3):572. doi: 10.3390/cancers12030572 (PMC7139631; doi:10.3390/cancers12030572)
Supplement: Supplementary file 1 [file cancers-12-00572-s001.pdf]

Supplementary Materials: Development and Validation of a Clinically Relevant Workflow for MR-Guided Volumetric Arc Therapy in a Rabbit Model of Head and Neck Cancer

Eftekhar Rajab Bolookat, Harish Malhotra, Laurie J. Rich, Sandra Sexton, Leslie Curtin, Joseph A. Spernyak, Anurag K. Singh and Mukund Seshadri

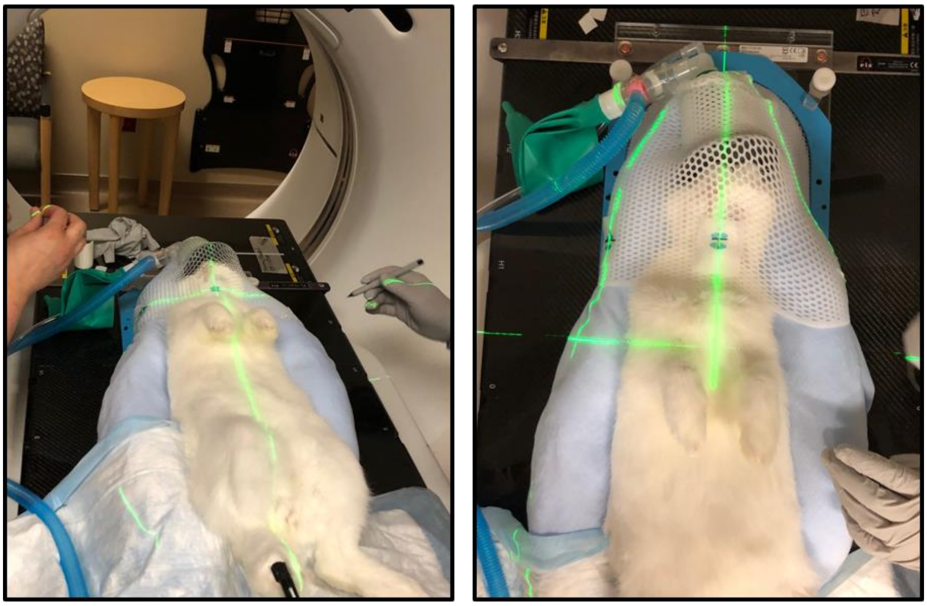

Figure S1. Rabbit set up and treatment planning. Anesthetized rabbits were positioned supine on the CT table and three fiducial markers were placed on the neck at the laser intersections to serve as reference points for treatment planning as shown above in the digital photograph.

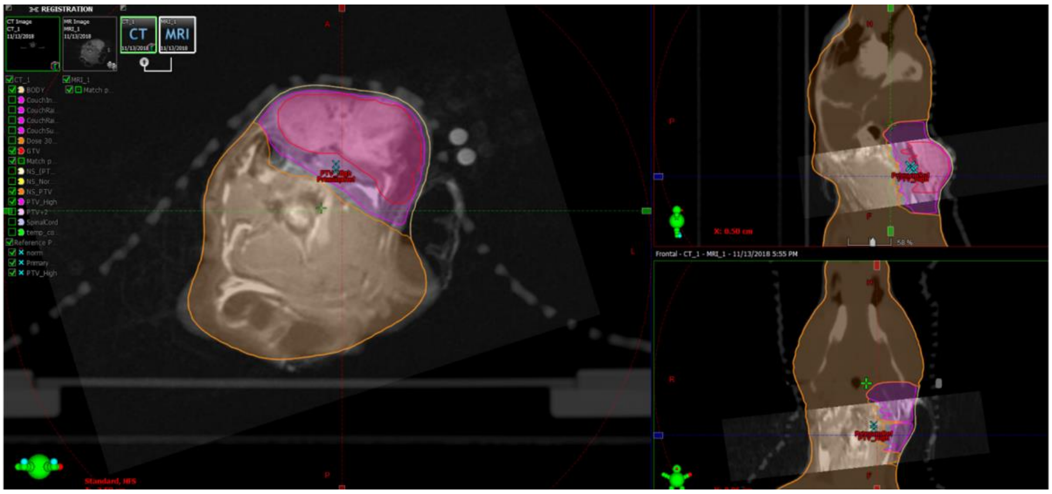

Figure S2. Registration of CT and MRI on treatment planning system. The MRI scan was fused to CT scan using manual registration module in Eclipse treatment planning system. GTV (red outline) was delineated on the overlaid CT-MRI axial section (left), sagittal (top right) and coronal (bottom right) cross sections were reconstructed by treatment planning system.

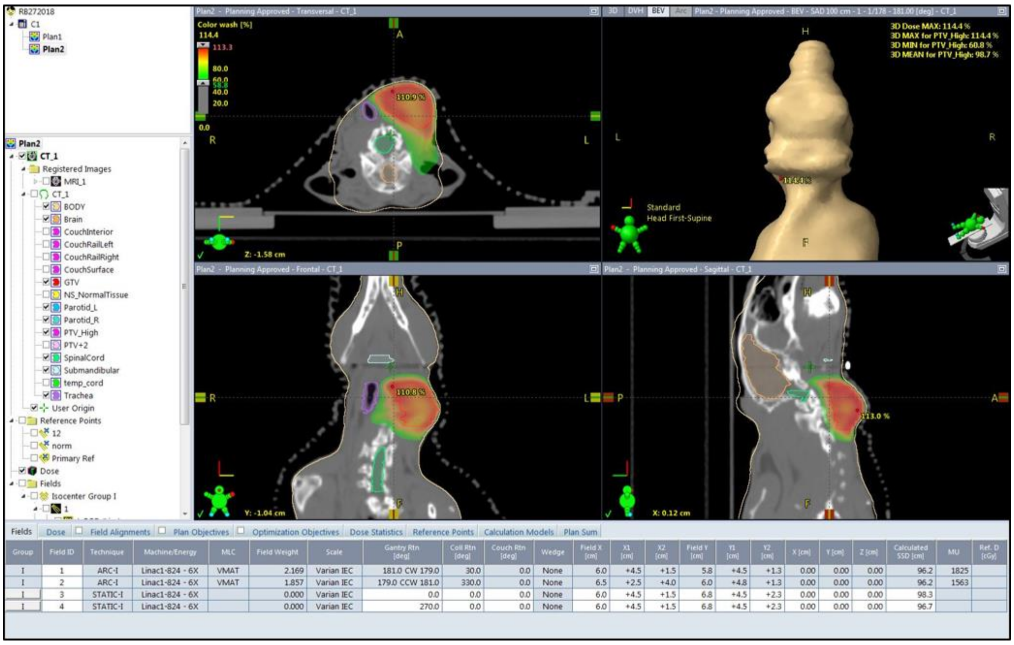

Figure S3. Treatment planning interface. Images of a VX2 tumor bearing rabbit contoured for the planning (left column), dose distribution on axial, coronal and axial views (right) and the characteristics of treatment fields including, gantry, collimator, field size, monitor unit, are shown in the bottom.

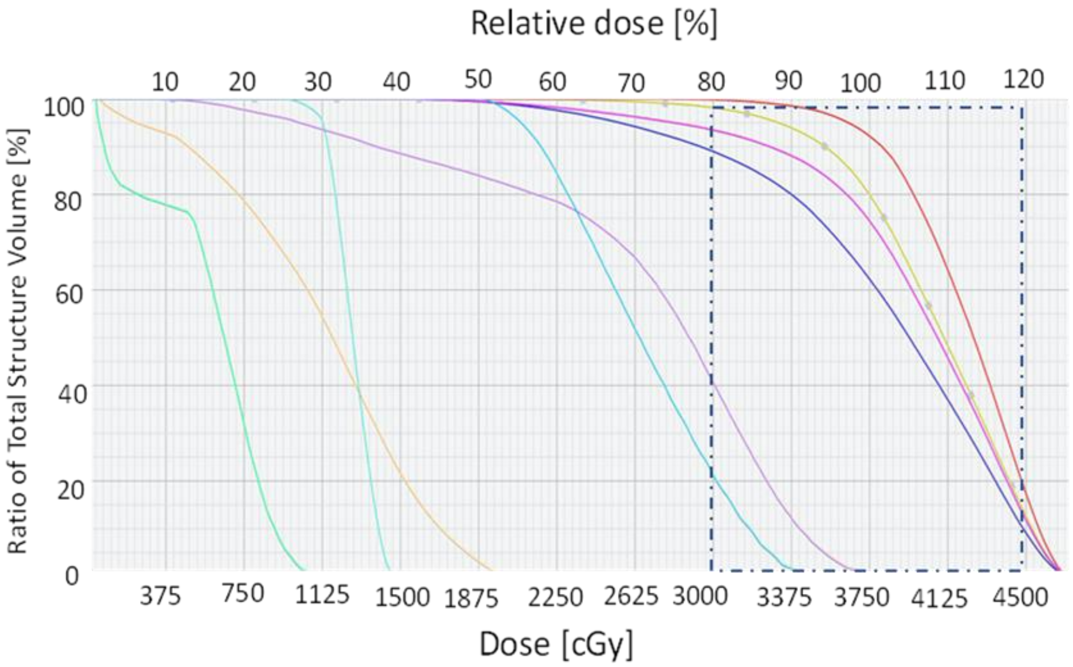

Figure S4. Dose volume histogram of CT-based treatment plans. Dose volume histogram (DVH) showing radiation dose to CT-GTV (yellow), CT-PTV (dark blue), MRI-GTV (red) and MRI-PTV (pink). All four regions are shown within the box in blue. Organs at risk included spinal cord (green), brain (orange), light blue (right parotid), cerulean blue (left parotid) and trachea (purple).

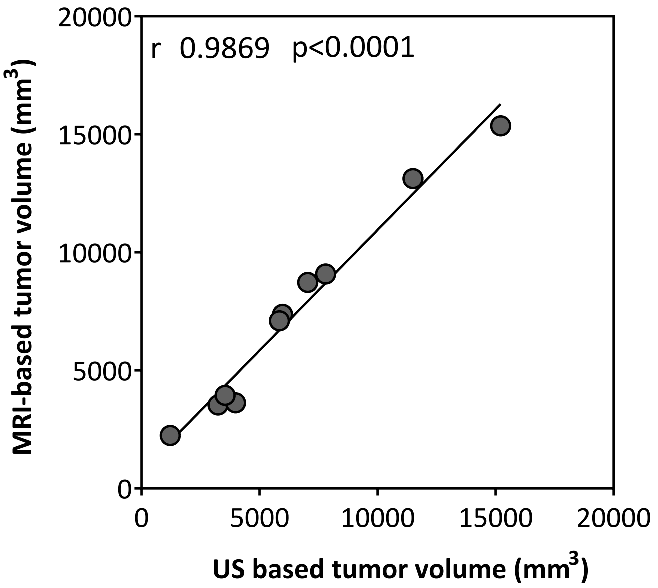

Figure S5. Correlation between MR and US based tumor volume**.** Plot shows correlation between MR and B-mode US based volumes of VX2 tumors at baseline. A strong correlation was seen between tumor volumes calculated using the two imaging methods. Long-term tumor response to RT was assessed using longitudinal US examinations over a 8-week period.
